# Cyclopedia of the Practice of Medicine, Vol. XIII

**Published:** 1878-12

**Authors:** 


*Cyclopœdia of the Practice of Medicine*, Edited by D. H. VON ZIEMSSEN, Vol. XIII. Diseases of the Spinal Cord and Medulla Oblongata. By Prof. Wilhelm Heinrich Erb, of Heidelberg, Baden. Albert H. Buck, M. D., Editor of American Edition. New York: Wm. Wood & Co.

This large sized volume is the work of one author alone. We have been impressed while reading it, with a feeling of admiration for the degree of success in treatment of the subject, which has been attained. When the amount of labor which is expended in gathering material, and the frequent exercise of good judgment necessary in recognizing truth amont conflicting statements of observers is considered, it seems remarkable that so many generalizations should have been arrived at and that there should be so great a degree of plainness in expression in the statements of the volume.

The front part of the work is occupied with a general consideration of the anatomy, physiology, symptomatology, etiology, diagnosis and therapeutics of the spinal cord and its diseases. This part is especially attractive and valuable. The centres of co-ordination are considered to be in the brain only, and are supposed to be in the corpora quadrigemina, the thalami optici and the cerebellum; no such centres seem to exist in the cord although combined reflex movements may be evoked therefrom.

A *tonus* of the vascular muscles, the author says, seems to be proved. This is maintained by vaso-motor nerves kept in a slight constant excitement by centres demonstrated in the medulla oblongata and the spinal cord. After the removal of these centres, peripheral ganglionic apparatuses have the power also of keeping up the tone of the vessels.

For practical guidance in recognizing the affections which may present in diseases of the cord, the author gives the following statement of principles, deduced from physiological and pathological experience:—

1. Section or limited affection of the posterior columns destroys the sense of touch in parts situated behind the point of injury, but leaves the sense of pain. 2. Disturbance of the conductive power of the gray substance for a limited longitudinal extent suspends the sense of pain, but leaves that of touch (analgesia). 3. Disease or destruction of the entering posterior root-fibres (or the network of fibres directly formed by them) must impair the sense of touch equally with that of pain and the other classes of sensation. 4. Injury or disease of the posterior columns at the level of the lumbar cord leads to a diminution of the sense of touch at the anus, perineum, etc., while the sensibility and motility of the lower extremities remain unimpaired; the same lesions in the lateral columns of the lumbar cord have the same effect upon the lower extremities as those of the posterior columns in the dorsal and cervical medulla. 5. When the gray substance is partially destroyed in the transverse direction, and the posterior columns are also affected, the conduction of sensory impressions is retarded, in a degree proportional to the smallness of the transverse piece of gray matter that remains. But if the conductive power of the posterior columns is retained, this retardation appears to extend only to the sensation of pain, while the conduction of the sensation of touch takes places with normal rapidity. 6. Destruction of the entire extent of the posterior columns (inclusive of the sensitive root-fibres passing through them) must be followed by anæsthesia of a corresponding extent. 7. Limited destruction of the entire transverse extent of the posterior columns and of the gray substance is followed by complete anæsthesia of the portions of the body lying posteriorly, and weakness of motion or partial paralysis. 8. An *irritation* affecting a limited longitudinal extent of the posterior columns (inflammation, hyperæmia, etc.), produces a spontaneous pain in only those roots which traverse the diseased spot (girdle-pain; subjective formations of touch (formication, prickling, numbness, sensation of heat and cold) and some degree of hyperæsthesia occur in the parts situated posteriorly. 9. *A lesion producing paralysis*, affecting the posterior columns in the same way, gives rise to a girdle of complete insensibility, correspondiag to the district supplied by the paralyzed nerve-roots; below this girdle the so-called sensations of touch are absent, or greatly impaired; the sensation of pain is retained, but is badly localized. 10. If an affection which at first irritates, and afterwards paralyzes, progresses upwards, the painful girdle travels upwards, and leaves behind it a girdle of anæsthesia which gradually increases in width; in the parts situated behind this the touch is gone, but subjective impressions of touch (formication, numbness, etc.) may be present. 11. When the power is unimpaired, and a girdle of pain without aberration of the sense of touch is present, then only the nerve-roots, within or without the cord, are affected. 12. In diseases of the posterior columns and the gray substance, the parts behind the diseased portion may experience only changesin the sense of touch, without any excentric pains. (?) If the latter occur, they point to an implication of those nerve-roots which are situated further back. 13. Disorganization of an anterior and a lateral column and of the greatest part of the gray substance produces paralysis of the same side. 14. Destruction of the anterior (and lateral) columns in their entire transverse section (inclusive of the motor nerve-roots passing through them) is followed by a paralysis of corresponding extent. 15. Limited destruction of the entire transverse section of the anterior (and lateral) columns and of the gray substance is followed by complete paralysis, analgesia, but retention of the sense of touch. 16. Disease of the antero-lateral columns and the kinesodic substance alone produces paralysis without lesion of sensibility. 17. Disease of the motor ganglia, into which the motor roots first enter, produces paralysis in the region of the related nerves, without disturbance of sensibility, but with trophic disturbances. 18. Affections of the antero-lateral columns and the corresponding gray substance produce contracture or convulsions *only* in the muscles immediately dependent on the diseased spot and its motor roots; but contractures of muscles supplied by the roots given off behind the affected spot are not produced (?). 19. Slight pressure on the cord may bring on paralysis of the extensors and secondary contractures in flexion, but this is never severe. 20. Contractures and convulsions of the lower extremeties also occur in affections of the segments of the cord above the lumbar region; they are then a consequence of an implication of the posterior columns, and arise reflexly. In the same way, in diseases of the posterior columns, spasmodic symptoms occur in the parts situated near the head. 21. Disorganization of the entire gray substance to a considerable distance must be followed by anæsthesia and paralysis in the posterior part of the body; if the lesion is limited to one place, the sensory and motor paralysis may be partial. 22. If the movements of respiration are entirely intact in an affection of the cervical cord which paralyses the extremities and trunk, then the lateral columns are not involved. 23. Conditions of irritation in the cervical medulla will produce dilatation of the pupil; paralytic conditions, contraction. 24. Unilateral lesion of the cord is followed by almost total paralysis and increased sensory excitability on the injured side, with very slight disturbances of motion and loss of sensibility on the opposite side. 25. Complete compression or division of the spinal cord exaggerates the reflex acts in the region lying posteriorly to the lesion. 26. In limited destruction of the dorsal medulla, the reflex acts which are performed through the lumbar cord (evacuation of urine and fæces, tonus, etc.) go on with very little alteration; only they can no longer be modified by the will. 27. The nutrition of peripheral parts (muscles, nerves, bones, joints, skin, etc.) remains intact in the various diseases of the spinal cord, in proportion as the gray substance remains normal.

The subjects of wounds, concussion and compression of the cord are carefully presented. The section on spinal irritation is introduced with a statement of the former abuse to which this term was subject and of the comparative disuse into which it has, as a consequence, fallen. The author, however, feels justified in using this term for a class of cases considerable in number, which are described by it, and which those who have large practical experience are not able to refer either to hysteria, neuralgia, intermittent fever, disease of internal organs, or to class among ordinary spinal diseases.

The portion which treats of *tabes dorsalis* or locomotor ataxy is worthy careful consideration. The course of the disease; the amaurosis and severe pains which may precede the ataxic symptoms by a considerable interval and the succeeding train of symptoms, are thoroughly described. The ataxia in diseases of the cord has not yet been satisfactorily referred to disease of the posterior or of the lateral columns alone. In *tabes*, sclerosis of the posterior columns is not the only condition found, but in the majority of cases, perhaps in all, the process extends beyond the posterior columns to the lateral columns and the posterior horns of the gray matter, often involving them to a very considerable extent.

In the main, the volume is worthy of welcome as a plain and thorough practical treatise, and a desirable acquisition to our literature.

